# Dairy Sheep Played a Minor Role in the 2005–2010 Human Q Fever Outbreak in The Netherlands Compared to Dairy Goats

**DOI:** 10.3390/pathogens10121579

**Published:** 2021-12-03

**Authors:** Piet Vellema, Inge Santman-Berends, Frederika Dijkstra, Erik van Engelen, Marian Aalberts, Carlijn ter Bogt-Kappert, René van den Brom

**Affiliations:** 1 Department of Small Ruminant Health, Royal GD, P.O. Box 9, 7400 AA Deventer, The Netherlands; c.t.bogt@gdanimalhealth.com (C.t.B.-K.); r.vd.brom@gdanimalhealth.com (R.v.d.B.); 2 Department of Research and Development, Royal GD, P.O. Box 9, 7400 AA Deventer, The Netherlands; i.santman@gdanimalhealth.com (I.S.-B.); e.v.engelen@gdanimalhealth.com (E.v.E.); m.aalberts@gdanimalhealth.com (M.A.); 3 Centre for Infectious Disease Control, National Institute for Public Health and the Environment (RIVM), P.O. Box 1, 3720 BA Bilthoven, The Netherlands; Frederika.dijkstra@rivm.nl

**Keywords:** *Coxiella burnetii*, caprine, sheep, goat, monitoring, surveillance, PCR, ELISA

## Abstract

Q fever is an almost ubiquitous zoonosis caused by *Coxiella burnetii*. This organism infects several animal species, as well as humans, and domestic ruminants like cattle, sheep and goats are an important animal reservoir of *C. burnetii*. In 2007, a sudden rise in notified human Q fever cases occurred in The Netherlands, and by the end of 2009, more than 3500 human Q fever patients had been notified. Dairy sheep and dairy goats were suspected to play a causal role in this human Q fever outbreak, and several measures were taken, aiming at a reduction of *C. burnetii* shedding by infected small ruminants, in order to reduce environmental contamination and thus human exposure. One of the first measures was compulsory notification of more than five percent abortion within thirty days for dairy sheep and dairy goat farms, starting 12 June 2008. After notification, an official farm inspection took place, and laboratory investigations were performed aiming at ruling out or demonstrating a causal role of *C. burnetii*. These measures were effective, and the number of human Q fever cases decreased; levels are currently the same as they were prior to 2007. The effect of these measures was monitored using a bulk tank milk (BTM) PCR and an antibody ELISA. The percentage PCR positive dairy herds and flocks decreased over time, and dairy sheep flocks tested PCR positive significantly less often and became PCR negative earlier compared to dairy goat herds. Although there was no difference in the percentage of dairy goat and dairy sheep farms with a *C. burnetii* abortion outbreak, the total number of shedding dairy sheep was much lower than the number of shedding dairy goats. Combined with the fact that Q fever patients lived mainly in the proximity of infected dairy goat farms and that no Q fever patients could be linked directly to dairy sheep farms, although this may have happened in individual cases, we conclude that dairy sheep did not play a major role in the Dutch Q fever outbreak. BTM monitoring using both a PCR and an ELISA is essential to determine a potential *C. burnetii* risk, not only for The Netherlands but for other countries with small ruminant dairy industries.

## 1. Introduction

Q fever is an almost ubiquitous, often occupational, zoonosis caused by *Coxiella burnetii*, which is able to infect several animal species, as well as humans [1,2]. Domestic ruminants like cattle, sheep, and goats are an important animal reservoir of *C. burnetii* [3,4,5,6]. In small ruminants, infections are usually not accompanied by clinical symptoms; however, abortions and stillbirths can occur, mainly during late pregnancy. Infected animals can shed the organism in faeces, milk and, mostly, in placental membranes and birth fluids [5,7,8,9]. During parturition of small ruminants, billions of bacteria from birth products become aerosolized, and transmission to humans occurs primarily through inhalation of aerosolised bacteria [5,10,11].

Q fever was first described in the early 1930s in abattoir workers in Australia [12], and rickettsial organisms were thought to play a role in infection [13]. Studies on Rocky Mountain spotted fever in America near Nine Mile Creek, Montana [14], demonstrated that the ‘Nine Mile agent’ was present in vacuoles of infected tissue culture cells and in developing chicken embryos and was able to cause infection in humans [15,16,17,18]. The American and Australian groups started working together and demonstrated that the Australian Q fever agent and the Nine Mile agent were in fact isolates of the same species, *Rickettsia diaporica* [16], *Rickettsia burneti* [5,19], later renamed as *C. burneti* [20] and finally *Coxiella burnetii*, a name that honours both Cox and Burnet as Q fever pioneers [21].

Since the first documented outbreaks, Q fever has been identified in countries all over the world. In Europe, human cases of Q fever were first reported from soldiers in the Balkan region in 1940 [22]. The World Health Organization reported its existence in 1955 in 51 countries on five continents, but not in New Zealand, Poland, the Scandinavian countries, and The Netherlands [23]. Today, this disease occurs worldwide, with the exception of New Zealand, where endemic Q fever has never been seen [24,25], although imported cases of human Q fever due to an infection acquired abroad cannot completely be prevented [26].

In The Netherlands, Q fever was diagnosed for the first time in humans in 1956 [27] and became a notifiable disease in 1978. Between 1978 and 2006, the average number of notifications per annum was seventeen. In 2007, a sudden rise in notified cases occurred, and this year, in hindsight, was the first year of the largest recorded community outbreak of Q fever, with more than 3500 confirmed human Q fever patients from 2007 to 2009 [28]. Two years earlier, in 2005, *C. burnetii* had been diagnosed for the first time as the cause of abortion on a dairy goat farm in The Netherlands, and between 2005 and 2009, this diagnosis was confirmed on 28 dairy goat and two dairy sheep farms [21]. Both animal species were suspected to have played a causal role in the human Q fever outbreak, and based on the fact that infected dairy goat farms were mainly located in the vicinity of human cases, it was concluded that dairy goats played a major role in human infections [11]. Because in outbreaks elsewhere sheep also played an important causal role [29], and abortion caused by *C. burnetii* had been confirmed on two dairy sheep farms in The Netherlands, much information has been collected about the role of dairy sheep. However, the results of these studies have not been described in relation to each other and in relation to the role of dairy goats in this outbreak.

This paper gives a coherent overview of the results of *C. burnetii* studies on Dutch dairy goat and dairy sheep farms, including implemented measures and the results of monitoring and surveillance, and concludes with recommendations for the most effective control of *C. burnetii* within the small ruminant dairy industry on a nationwide level.

## 2. Results

### 2.1. Historic Results

#### 2.1.1. Historic Information on Dairy Sheep and Dairy Goat Farming in The Netherlands

At the start of the Q fever outbreak in 2007, the number of breeding ewes in The Netherlands was estimated to be a little less than one million, of which only a small number were dairy sheep [21]. Dairy sheep have been present in The Netherlands for centuries [30] and are often kept on small scale farms. In 2007, commercial dairy sheep were kept on forty farms, and the number of animals per farm varied from less than fifty to almost one thousand; most of the farms kept one hundred to four hundred animals [21]. The dairy goat industry started after the introduction of the European milk quota system for dairy cattle in 1984. Initially, the average number of goats per dairy farm was around 200 [31]. In 2007, there were 350 farms, with an average of almost one thousand dairy goats per farm [32,33]. Total dairy goat milk production increased from almost zero to 150,000 tons annually in 25 years [29]. 

#### 2.1.2. Studies and Events on Dairy Goat and Dairy Sheep Farms

*C. burnetii* was diagnosed for the first time as a cause of abortion on a dairy goat farm in The Netherlands in 2005 [8], and until the end of 2009, *C. burnetii*–induced abortion waves were confirmed on 28 dairy goat and two dairy sheep farms [21]. The average number of goats and sheep per infected farm were 900 and 400, of which on average 20% and 5% of the pregnant animals aborted, respectively [21]. The rise in human Q fever cases was confirmed in 2007, although, it is plausible that cases occurred as early as 2005 [34]. The human Q fever outbreak started in the southeastern part of The Netherlands, in the same area where small ruminant abortion waves occurred. In response to the large human outbreak, *C. burnetii*–induced abortion was made a notifiable disease in June 2008 for dairy sheep and dairy goat farms with more than fifty small ruminants. Nine abortion outbreaks were confirmed between June 2008 and the end of 2009; however, since 2009, no cases of abortion caused by *C. burnetii* were reported in small ruminants [35]. 

Since 2007, several *C. burnetii*–related studies on sheep and goat farms have been performed. For instance, in 2008, the seroprevalence of *C. burnetii* infections was measured, based on *Brucella melitensis* surveillance samples that were compulsorily collected annually on 1450 small ruminant farms in The Netherlands. In total, 2.4% of the sheep and 7.8% of the goat samples were seropositive. Risk factors for seropositivity were the use of animals for dairy production, location of the premises in the southeastern part of The Netherlands, and testing during pregnancy or in the periparturient period [36].

In 2008, the prevalence of *C. burnetii* on the herd or flock level was measured by PCR testing of voluntarily submitted bulk tank milk (BTM) samples from 292 dairy goat and 16 dairy sheep farms, using a PCR with a described optimal cut-off value of 100 bacteria/mL. None of the samples from the dairy sheep farms (*n* = 16) and 96 (32.9%) samples from dairy goat farms tested PCR positive [37]. In October 2009, monthly BTM PCR testing became compulsory for professional dairy sheep and dairy goat farms with more than fifty adult animals, and this is still the case in 2021. Since May 2016, no PCR-positive BTM results have been confirmed [38].

The role of manure as a probable source of a human Q fever outbreak has been discussed [39]. To estimate this role in The Netherlands, decimal reduction times of the Nine Mile RSA 493 reference strain of *C. burnetii* in relevant matrices under experimental circumstances, and the impact of manure storage in dunghills on *C. burnetii* survival, were investigated [40]. Results indicated no association between the incidence of human Q fever cases and the dispersal of goat manure originating from farms with confirmed *C. burnetii* abortion waves in 2008 and 2009. These results are reinforced by the results of the study of Roest et al. [9], which confirmed that faeces are not an important source of environmental contamination. Although another Dutch study reported an association between land-applied goat manure and human Q fever cases [41], no correction was made in this study for the presence of infected farms in the region where manure had been distributed, and infected farms were misclassified given the data source used. Therefore, the role of land-applied manure in the Dutch Q fever outbreak seems highly unlikely.

After the rise in human Q fever patients in 2009, for the third year in a row, the Dutch government decided to cull all pregnant animals on dairy sheep and dairy goat farms with a BTM *C. burnetii* PCR-positive result [42]. At that time, the effect, under field conditions, of the unlicensed phase 1 vaccine Coxevac^®^ was unknown. To investigate the efficacy of the vaccine, vaginal mucus, uterine fluid, and milk samples were collected from culled vaccinated and unvaccinated dairy goats. This study showed that the prevalence and bacterial loads were significantly reduced in vaccinated animals compared with unvaccinated animals. These effects were most pronounced in animals during their first pregnancy. This indicated that vaccination reduced bacterial loads in the environment and subsequent human exposure to *C. burnetii* [43]. Since no dairy sheep had been vaccinated at that time, only dairy goats were included in this study.

#### 2.1.3. Measures Taken

The human Q fever outbreak led the Dutch government to implement several measures, aiming at a reduction of *C. burnetii* shedding by infected sheep and goats, in order to reduce environmental contamination and thus human exposure. One of the first measures was compulsory notification of an abortion outbreak on dairy sheep and dairy goat farms; starting on 12 June 2008, more than five percent abortion within thirty days must be reported [44]. After notification, an official farm inspection is carried out by an expert team consisting of a private veterinary practitioner, an official veterinarian of The Netherlands Food and Consumer Product Safety Authority, and a small ruminant specialist of GD; during the farm visit, samples are collected and laboratory investigations are performed, aimed at ruling out or demonstrating a causal role of *C. burnetii*. 

A voluntary vaccination campaign with the unlicensed inactivated phase I vaccine (Coxevac^®^, CEVA Santé Animale) started in the autumn of 2008. In 2009, vaccination became compulsory for dairy sheep and dairy goat farms in the southeastern part of The Netherlands. Starting in 2010, vaccination became mandatory for all dairy sheep and dairy goat farms with more than fifty animals, for small ruminants kept on farms with a public function, and for small ruminants participating in shows. A stringent hygiene protocol became mandatory in the first months of 2009 for dairy sheep and dairy goat farms [45]. 

After implementation of the above-mentioned measures in combination with a rise in seroprevalence in the human population as a result of exposure to *C. burnetii*, the number of human Q fever patients decreased; current levels are equivalent to those prior to 2007 [28,29,46]. Although it was not possible to measure the preventive effect of each individual measure, since measures were implemented more or less in the same period, the preventive effect of vaccination was the most pronounced [29]. 

Currently, most of the above-mentioned measures, such as *C. burnetii* infections on dairy sheep and dairy goat farms being reportable, compulsory vaccination, and hygiene measures, are still applicable in The Netherlands.

### 2.2. Current Results 

#### 2.2.1. Goat and Sheep Farming in The Netherlands

**Figure 1:**
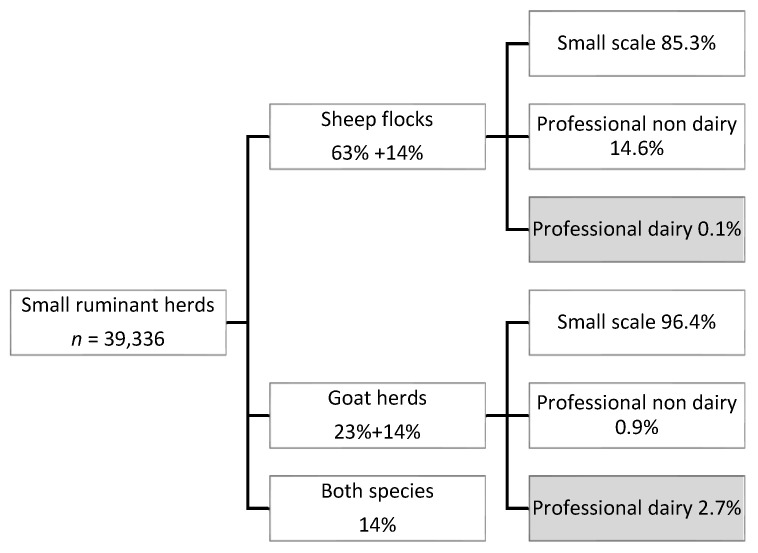
Number of small ruminant holdings in The Netherlands, percentages of flocks and herds containing sheep (63% only sheep, 14% sheep and goats), goats (23% only goats, 14% goats and sheep), or both species (14% both sheep and goats), and percentages of small scale and professional holdings per animal species.

The Dutch goat and sheep industry is relatively small, with 671,000 goats and 1,220,000 sheep in 2020. The total number of registered holdings with small ruminants in 2020 was 39,336. Of these holdings, most were classified as small scale holders (less than 32 heads), i.e., 85% of all sheep flocks and 96% of all goat herds (Figure 1). Only 0.1% of the sheep flocks (*n* = 35) and 2.7% of the goat herds (*n* = 400) were classified as a dairy herd (Figure 1). 

**Figure 2:**
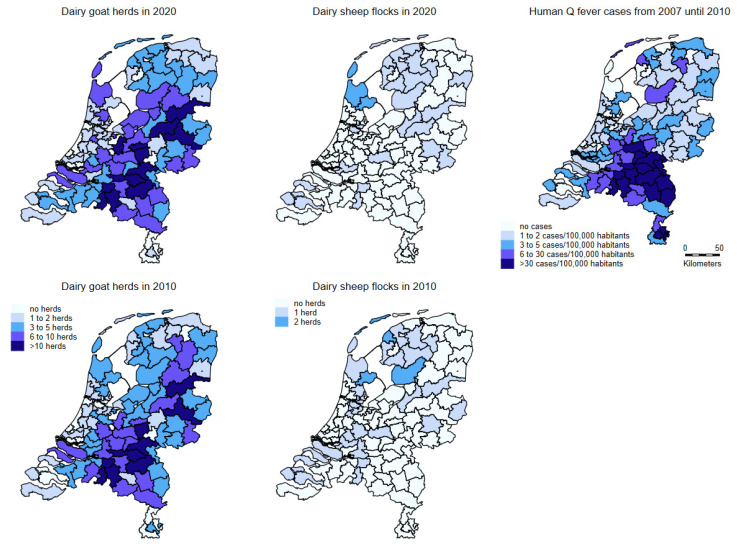
Densities of dairy goat herds (**left**; above in 2020 and below in 2010) and dairy sheep flocks (**middle**; above in 2020 and below in 2010). The right map shows the number of Q fever patients with confirmed status reported to the municipal health service from 1 January 2007 to 31 December 2010 per 100,000 population. All maps based on two digit postal code areas in The Netherlands.

In 2020, the dairy goat herds and sheep flocks housed on average 1411 and 493 heads of animals (median 1157 and 438), respectively. The dairy goat herds were dispersed throughout the country, with the highest numbers located in the southeastern part of The Netherlands. The dairy sheep herds are mainly located in the northern part of the country (Figure 2). The number of dairy goat farms increased in the last two decades, as well as the average number of goats per dairy goat farm. The number of dairy sheep farms and the number of sheep per farm has been more or less the same in that period. The number of dairy sheep per farm differs widely, from around fifty to around one thousand [47].

#### 2.2.2. Bulk Tank Milk Monitoring 

**Figure 3:**
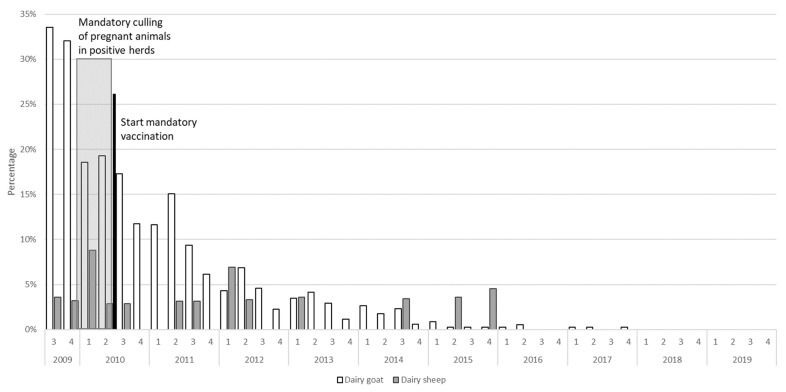
The percentage of *Coxiella burnetii* PCR-positive dairy goat herds (total population in 2020 *n* = 400) and dairy sheep flocks (total population in 2020 *n* = 35) in The Netherlands based on the bulk tank milk monitoring program between 2009 and 2020.

**Figure 4:**
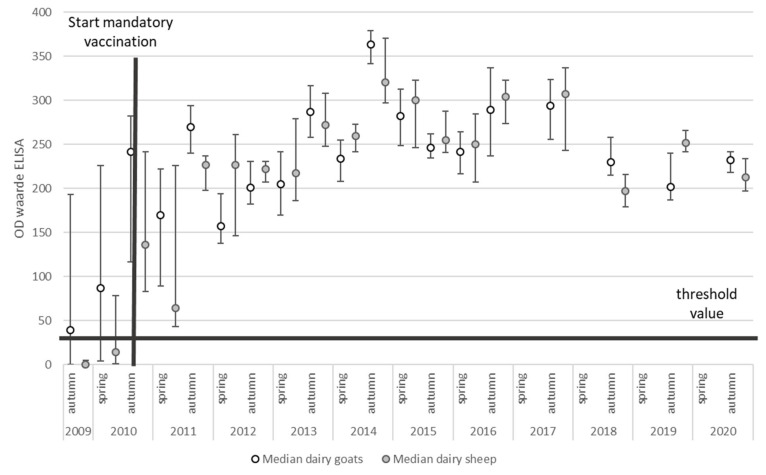
The median and interquartile range (IQR) of the SP% of *Coxiella burnetii* BTM samples of all dairy goat herds (*n* = 400) and dairy sheep flocks (*n* = 35) in The Netherlands between 2009 and 2020.

After implementation of risk-mitigating measures like mandatory vaccination and culling, the effect of these measures could be monitored using the PCR and antibody ELISA results from the BTM monitoring program (Figure 3 and Figure 4).

Descriptively, the percentage of dairy herds and flocks that tested PCR positive for the presence of *C. burnetii* appeared to decrease over time (Figure 3). The results of regression analysis indicate that dairy sheep flocks tested PCR positive significantly less often than dairy goat herds (IRR = 0.29, 95% CI: 0.13–0.69, *p*-value = 0.005). Additionally, after implementation of the risk mitigating measures, a significant decrease of PCR positive results occurred (IRR = 0.14, 95% CI: 0.05–0.44, *p*-value = 0.001).

The BTM antibody ELISA results showed an increase in mean SP% (sample-to-positive percentages) since the implementation of vaccination (Figure 4). Due to the small number of antibody-negative BTM samples, it was not possible to explore factors associated with having an antibody-positive BTM. When we evaluated factors that were associated with SP% in antibody-positive herds, it was observed that SP% increased over time, with an average increase in SP% of 3 per year (95% CI: 2–4, *p*-value < 0.001), and it was significantly higher in the autumn evaluation compared to the spring evaluation, with an average difference in SP% of 30 (95% CI: 27–34, *p*-value < 0.001). We did not observe a significant difference in SP% between sheep flocks and goat herds (*p*-value = 0.72).

#### 2.2.3. Human Notifications 

The geographical display of notified human Q fever patients (Figure 2) shows that most of the patients did not live in the area where the majority of dairy sheep were kept.

## 3. Discussion

Between 2005 and 2010, The Netherlands faced the largest recorded community outbreak of Q fever, and dairy goats and dairy sheep were regarded as the main sources of *C. burnetii* shedding. A package of measures was implemented, aiming to determine the presence of shedding animals and to reduce environmental contamination. Examples of measures taken were hygiene measures, restrictions for visitors, clothing protocols for professional visitors, compulsory removal of foetuses and placental membranes for rendering, measures with regard to the handling of manure, compulsory vaccination, an expansion ban for all dairy sheep and goat farms, and animal movement restrictions and a breeding ban for infected farms [29]. These measures seemed highly effective, but because several measures were implemented at the same time, the effect of these measures cannot be estimated separately. Only vaccination has been demonstrated, taken on its own, to be effective [43]. 

At the start of the human Q fever outbreak, it was not clear which criteria could be used to identify the small ruminant farms that posed a risk to humans. Infections with *C. burnetii* do not always cause abortion in sheep and goats [9,48], and shedding can occur after a seemingly normal parturition [9]. Therefore, it was decided to start a BTM program, using a *C. burnetii* PCR. This appeared to be a very sensitive method for detecting only a few shedding lactating animals on large dairy goat farms [49]. In this way, farms with and without increased abortion rates were detected. Initially, this program was intended to declare farms free from *C. burnetii*, but since the end of 2009, PCR-positive farms were declared infected, and pregnant animals on these farms were culled. Even though this measure prevented the parturition and shedding of many potentially infected animals, it remains under debate, since *C. burnetii* is an ubiquitous bacterium that can be shed by several animal species and can cause long-term environmental infection [50]. Furthermore, no distinction was made between vaccinated and non-vaccinated animals, while it was later shown that vaccination is very effective [43]. The correlation between *C. burnetii* shedding in milk and occurrence of abortion is unclear, but does not seem to be very high. Furthermore, shedding of *C. burnetii* only in milk does not pose a high risk to humans, especially if milk is pasteurised. However, it was shown that persons involved in the culling of the pregnant animals at BTM PCR-positive farms, despite the use of personal protective equipment, contracted Q fever, which shows that such farms can pose risk to humans, even without high abortion rates [51]. 

In The Netherlands, BTM ELISA was used to monitor whether antibody responses were obtained after vaccination. Most of the ELISAs were conducted in the period in which vaccination was mandatory. Therefore, the number of antibody-negative BTM samples was low. In situations where no vaccination was applied, a positive ELISA was indicative of a previous *C. burnetii* infection. Thus BTM monitoring using both a PCR and an ELISA is useful to determine a potential *C. burnetii* risk.

Abortions due to *C. burnetii* result in shedding of large amounts of bacteria in the environment. Vaccination with a phase 1 vaccine is very effective, especially in animals that are vaccinated before their first pregnancy [43]. In The Netherlands, vaccination started voluntarily in 2008, voluntarily or compulsory in 2009 depending on the region, and has been compulsory nationwide since 2010 for dairy goat and dairy sheep farms with more than fifty animals, for farms with a public function, and for goats and sheep visiting shows. Since compulsory vaccination started, no cases of abortion caused by *C. burnetii* have been detected on these farms. BTM surveillance showed that the number of dairy goat and dairy sheep farms shedding *C. burnetii* in milk decreased gradually, reaching zero in 2015 for dairy sheep farms and in 2016 for dairy goat farms; the initially PCR-positive cases in 2017 were not confirmed, probably because intermittently shedding animals sometimes only do so for a very short period of time. Based on this information, vaccination aiming at controlling the shedding of *C. burnetii* in small ruminants has to be performed for at least eight years, possibly until the last animals have left the farm that had been pregnant before vaccination started. Despite the advantages of vaccination, there are also some disadvantages of this vaccination regime, such as high financial costs and reported adverse reactions, such as a temporary drop in milk yield; especially in sheep, farmers regularly report deaths in animals that have been vaccinated more than three times. In sheep, the initially recommended vaccination dose was 1 mL, and the results of monitoring have shown that this dose has been effective, since no cases of abortion caused by *C. burnetii* have been found in dairy sheep flocks since vaccination started, and no shedding of *C. burnetii* has been demonstrated in milk from dairy sheep farms since 2015. Additionally, annual BTM ELISA results in samples from dairy sheep farms vaccinated with a dose of 1 mL showed a high humoral response [37]. Therefore, based on the results of our studies, the need to use a 2 mL dose as prescribed for cattle and goats is questionable.

From 2005 up to and including 2009, abortions caused by *C. burnetii* were confirmed on 28 dairy goat farms, mainly located in the southern part of the country, and on two dairy sheep farms, one in 2006 in the south and another in 2008 in the northern part of the country [21]. In that period, the total number of locations where small ruminants were kept was estimated to be around 40,000, and only on 28 out of 350 (8%) dairy goat farms, and two out of forty (5%) dairy sheep farms, abortions caused by *C. burnetii* have been confirmed. No cases of abortion caused by *C. burnetii* have ever been confirmed on the remaining non-dairy farms, while the number of submitted foetuses and placentas were larger for non-dairy farms compared to dairy farms. This is in agreement with the fact that for both sheep and goats, dairy was a risk factor for seropositivity [36]. 

In several studies, mainly case descriptions, sheep have been appointed as a source of human Q fever outbreaks [29,52,53,54,55,56]. In the study of de Lange et al. [54], the seroprevalence in dairy sheep farm residents (66.7%) was higher compared to non-dairy sheep farm residents (51.3%). However, based on the outcome of several studies during this Dutch human Q fever outbreak [57,58,59], goats were found to be a far more likely source compared to sheep. It often turns out afterwards that, during an outbreak, more samples should have been collected and stored properly. If this had been done during the Dutch Q fever outbreak, it would have been possible to perform in-depth analyses aiming at confirming or denying possible links between human and animal *C. burnetii* isolates. Molecular epidemiology of clinical samples has been performed [60,61,62,63], and the first study [60] demonstrated that farm animals and humans were infected by different but apparently closely related genotypes. Roest et al. [61], using a 10-loci multilocus variable-number tandem-repeat analysis panel (MLVA typing), identified one predominant genotype among goats and sheep throughout the entire affected Q fever area, and another study [62] concluded that multiple different but closely related genotypes were involved. Tilburg et al. [63] confirmed that sheep and goats, and not cattle, were the source of human Q fever in The Netherlands and concluded that goats were the most probable source. However, the relatively few isolates from sheep could also be linked to humans, and it cannot be excluded that goats not only infected humans but also sheep. Increasing understanding of the genetic diversity of *C. burnetii* [64,65] supports our hypothesis that a lack of sheep samples in genotyping studies hinders conclusive observations.

Differences in shedding exist between infected cattle, sheep, and goats [66,67,68], but it is not clear what the differences between these animal species mean; however, as far as we know, Q fever outbreaks in humans related to cattle have not been documented. The study of Rodolakis et al. [68] focuses on shedding in milk, but milk is usually not a source of environmental contamination. Because both infected sheep and goats have been demonstrated to shed large amounts of *C. burnetii* during abortion or parturition [52,57,59], with the current knowledge, it is not possible to know which of the two animal species excretes the largest amount of this bacterium.

Although there was no difference in the percentage of dairy goat and dairy sheep farms with a confirmed *C. burnetii* abortion outbreak, the total number of shedding dairy sheep per farm was much lower than the number of shedding dairy goats, as the average number of goats on infected farms was 900, from which on average 20% of the pregnant animals aborted; the average number of sheep on the two infected sheep farms was 400, with an abortion rate of around 5% [29]. Results of BTM monitoring indicated that the percentage of dairy herds and flocks that tested PCR positive for the presence of *C. burnetii* decreased over time, but dairy sheep flocks tested PCR positive significantly less often compared to dairy goat herds. Combined with the fact that confirmed notified Q fever patients mainly lived near infected dairy goat farms with a history of abortion, and no Q fever patients could be linked directly to dairy sheep farms, although that does not exclude that this may have happened in individual cases, we conclude that dairy sheep did not play a major role in the Dutch Q fever outbreak.

BTM monitoring using both a PCR and an ELISA was and still is essential to determine the potential *C. burnetii* risk and to monitor the situation in the field. Additionally, increased numbers of abortions on small ruminant farms are notifiable, and based on vaginal mucus sampling, give insight into the Q fever status on these farms.

## 4. Materials and Methods

### 4.1. Historic Results

#### 4.1.1. Historic Information on Dairy Sheep and Dairy Goat Farming in The Netherlands

Data relating to the demographic situation of dairy sheep and dairy goats over the years were received from The Netherlands Enterprise Agency (Rijksdienst voor Ondernemend Nederland; RVO), which supervises the official database for identification and registration of sheep and goats in The Netherlands. The raw data from RVO are sent to IntoFocus Data Transformation Services (IDTS), which encrypts all variables in the data that might link the data back to the original source, such as the farm of origin or animal identification. To ensure that data from different organizations can be combined for analysis, a corresponding encryption code is used for all datasets.

#### 4.1.2. Studies on Dairy Goat and Dairy Sheep Farms

For the first cases of *C. burnetii*–induced abortion on dairy goat farms in The Netherlands, the diagnosis was confirmed on the basis of immunohistochemistry after necropsy of foetuses and placenta [8]. Lack of a gold standard for the detection of *C. burnetii* meant that, for subsequent studies, different techniques were used to study the presence of infection in sheep and goats in The Netherlands. These techniques also included commercially available RT-PCR and ELISA techniques, applied on both the herd and individual level. In addition to information from RVO, publicly available information regarding the *C. burnetii* status of farms was used from The Netherlands Food and Consumer Product Safety Authority (Nederlandse Voedsel- en Warenautoriteit; NVWA), the official organization dealing with notifiable diseases. Royal GD provided multiple data: data from their customer relationship management system, pathology data, and the results of the compulsory *C. burnetii* bulk tank milk monitoring program. 

### 4.2. Current Results 

#### 4.2.1. Goat and Sheep Farming in The Netherlands

Data relating to the demographic situation of dairy sheep and dairy goats were received from RVO.

#### 4.2.2. Bulk Tank Milk Monitoring 

In The Netherlands, a *C. burnetii* monitoring program based on BTM testing is in place to monitor and control infections. A complete description of this program can be found in van den Brom et al. [42]. This program started in October 2009, and until July 2017, BTM samples from all dairy sheep and dairy goat farms were tested twice a month using a PCR. After that, the frequency for PCR-negative farms was reduced outside the lambing season, from July up to November, to once a month, and later to once a month year round. Twice a year, in spring and in autumn, BTM was also tested for the presence of antibodies, using an indirect ELISA, and since July 2017, this has been reduced to once a year in autumn. ELISA results are evaluated in relation to the compulsory vaccination, which must be performed annually within a year after the last vaccination, and for lambs and kids, before the start of the breeding season.

#### 4.2.3. PCR Testing

BTM samples were tested for *C. burnetii* by PCR [42]. DNA was extracted using an AM1840 MagMAX™ Total Nucleic Acid Isolation Kit (Thermo Fisher Scientific, Waltham, MA, USA) according to the manufacturer’s instructions. Until September 2017, *C. burnetii* was detected using the TaqVet *Coxiella burnetii* Absolute Quantification kit (LSI, Lissieu, France) according to the manufacturer’s instructions. A sample was considered *C. burnetii* positive when more than 100 bacteria/mL were detected, using the calibration series of the manufacturer. 

From September 2017, after both PCRs were validated against each other, and using a standard to perform quantification, *C. burnetii* was detected using a *C. burnetii* KASP PCR based on the IS1111a gene, the same gene as in the previously used TaqVet PCR. The following primers were used: forward primer 5′-GAAGGTGACCAAGTTCATGCTGCGGCAATGTGATGTTAAGGAC-3′ and reverse primer 5′-ACGGGCGCCATGAATCAATA-3′. The real-time PCR was performed on an ABI7500 fast thermal cycler (Thermo Fisher Scientific, Landsmeer, The Netherlands) using the KaspRT reagent (LGC Genomics, Hoddesdon, UK) and the following protocol: initial denaturation for 15 min at 95 °C, followed by 45 cycles of denaturation for 20 s at 95°C and annealing and extension for 60 s at 57 °C (fast modus). Data were analyzed (with ROX) using a Delta Rn threshold of 0.2 for the FAM signal and baseline normalization based on PCR cycles 3 to 15. Based on the calibration series, samples with more than 100 bacteria/mL were regarded positive for *C. burnetii*. 

#### 4.2.4. ELISA Testing

BTM samples were tested for *C. burnetii* antibodies using the indirect ELISA PrioCHECK Ruminant Q fever Ab plate kit (Thermo Fisher Scientific), previously called Ruminants Serum Q fever LSI kit. ELISA was performed according to the manufacturer’s instructions, and results were expressed as sample-to-positive percentages (SP%), calculated using the formula: SP% = (OD_sample_ − OD_negative control_)/(OD_positive control_ − OD _negative control_) × 100%, where OD = optical density. Samples with an SP% > 30% were considered positive for *C. burnetii* antibodies [37].

#### 4.2.5. Human Notifications

In The Netherlands, attending physicians and microbiology laboratories are legally obliged to report any human Q fever diagnosis to the local municipal health service [28]. The number of reported Q fever patients with confirmed status in the period 1 January 2007 through 31 December 2010 per 100,000 inhabitants is shown in Figure 2, based on two digit postal code areas in The Netherlands.

#### 4.2.6. Analysis

For this study, data relating to the complete population of small ruminants in The Netherlands were available from RVO and GD. Because this study focused on dairy sheep flocks and dairy goat herds, data from non-dairy herds were removed. From the dairy herds that remained, descriptive statistics such as herd size and location were graphically presented for both sheep flocks and goat herds. 

PCR and ELISA results from the BTM monitoring program over the period 2009–2020 were graphically presented, stratified to dairy sheep flocks and dairy goat herds. To evaluate whether risk-mitigating measures were followed by a reduction in the number of PCR-positive herds in time, a negative binomial regression model in Stata^®^ version 17 was applied. In this model, the number of PCR-positive herds per measurement and per herd type (sheep or goat) were included as dependent variables, and the number of tested herds per measurement and mitigating measures were included as independent variables. Most of the ELISAs were conducted in the period in which vaccination was mandatory, which resulted in positive ELISA results. Therefore, the number of antibody-negative BTM samples was low. As a consequence, it was difficult to evaluate the association between ELISA results (positive versus negative) and herd type. In 2008, the initially recommended vaccination dose for sheep was 1 mL and for goats 2 mL, based on the instruction leaflet of this vaccine, which was initially authorised under ‘exceptional circumstances’, meaning that, at that time, is was not possible to obtain complete information about Coxevac^®^ [69]. Because of this difference in vaccination dose between goats and sheep, we were interested to see if this difference was reflected in differences in ELISA SP%. For this analysis, only SP% of ELISA positive test results (SP% > 30%) were selected, which were normally distributed. Using a multilevel linear regression model in Stata^®^ to correct for repeated measures within a herd, we evaluated the association between SP% of ELISA positive herds and herd type and season. 

## 5. Conclusions

This paper provides an overview of the results of *C. burnetii* studies on Dutch dairy goat and dairy sheep farms, the implemented measures, and the results of monitoring and surveillance, over more than a decade. Initially, dairy goats and dairy sheep were mentioned as the most likely source of infection for humans. We conclude that dairy sheep did not play a major role in this human Q fever outbreak. We also conclude that a combination of measures, but especially vaccination of dairy sheep and dairy goats, resulted in an effective control of human Q fever cases in a densely populated, developed country like The Netherlands.

BTM monitoring using both a PCR and an ELISA was and still is essential to determine the potential *C. burnetii* risk, not only for The Netherlands but also for other countries with small ruminant dairy industries. 

## Data Availability

An overview of results as presented in this paper is published in the small ruminant half-yearly monitoring and surveillance reports (Rapportage Monitoring Diergezondheid Kleine Herkauwers). The remaining data presented in this study are available on request from the corresponding author. These data are not publicly available due to privacy reasons.
